# Supplementary material for: Editorial commitment to trust and integrity in science: implications for pain and anaesthesiology research*
Source: Anaesthesia. 2025 Jul 1;80(9):1032–9. doi: 10.1111/anae.16655 (PMC12351217; doi:10.1111/anae.16655)
Supplement: Supplementary file 1 — Appendix S1. Details of editorial affiliations [file ANAE-80-1032-s001.docx]

**Appendix S1. Details of editorial affiliations**

Matthew Wiles, Editor-in-Chief, *Anaesthesia*;

Jaideep Pandit, Editor-in-Chief, *Anesthesia & Analgesia*;

Hugh Hemmings, Editor-in-Chief, *British Journal of Anaesthesia*;

Stephan K, W. Schwarz, Editor-in-Chief, *Canadian Journal of Anesthesia*;

Joel Katz, Editor-in-Chief, *Canadian Journal of Pain*;

Dennis Turk, Editor-in-Chief, *Clinical Journal of Pain*;

Marc Van de Velde, Editor-in-Chief, *European Journal of Anaesthesiology*;

Didier Bouhassira, Editor-in-Chief, *European Journal of Pain*;

Tony Yaksh, Editor-in-Chief, *Frontiers in Pain Research*;

Michael Schatman, Editor-in-Chief, *Journal of Pain Research*;

Theodore Price, Editor-in-Chief, *Neurobiology of Pain*;

Karen Davis, Editor-in-Chief, *PAIN*;

Robert Hurley, Editor-in-Chief, *Pain Medicine*;

David Yarnitsky, Editor-in-Chief, *PAIN Reports*; and

Tonya Palermo, Editor-in-Chief, *The Journal of Pain*.
